# Mitochondrial aconitase 1 regulates age‐related memory impairment via autophagy/mitophagy‐mediated neural plasticity in middle‐aged flies

**DOI:** 10.1111/acel.13520

**Published:** 2021-11-19

**Authors:** Yun‐Ho Cho, Gye‐Hyeong Kim, Joong‐Jean Park

**Affiliations:** ^1^ Department of Physiology Korea University College of Medicine Seoul Republic of Korea

**Keywords:** aconitase, age‐related memory disorders, autophagy, mitochondria, mitophagy, neural plasticity

## Abstract

Age‐related memory impairment (AMI) occurs in many species, including humans. The underlying mechanisms are not fully understood. In wild‐type *Drosophila* (*w^1118^*), AMI appears in the form of a decrease in learning (3‐min memory) from middle age (30 days after eclosion [DAE]). We performed *in vivo*, DNA microarray, and behavioral screen studies to identify genes controlling both lifespan and AMI and selected mitochondrial Acon1 (*mAcon1*). *mAcon1* expression in the head of *w^1118^* decreased with age. Neuronal overexpression of *mAcon1* extended its lifespan and improved AMI. Neuronal or mushroom body expression of *mAcon1* regulated the learning of young (10 DAE) and middle‐aged flies. Interestingly, acetyl‐CoA and citrate levels increased in the heads of middle‐aged and neuronal *mAcon1* knockdown flies. Acetyl‐CoA, as a cellular energy sensor, is related to autophagy. Autophagy activity and efficacy determined by the positive and negative changes in the expression levels of Atg8a‐II and p62 were proportional to the expression level of mAcon1. Levels of the presynaptic active zone scaffold protein Bruchpilot were inversely proportional to neuronal mAcon1 levels in the whole brain. Furthermore, mAcon1 overexpression in Kenyon cells induced mitophagy labeled with mt‐Keima and improved learning ability. Both processes were blocked by *pink1* knockdown. Taken together, our results imply that the regulation of learning and AMI by mAcon1 occurs via autophagy/mitophagy‐mediated neural plasticity.

## Graphical abstract

As aging progresses, the expression of the TCA enzyme mAcon1 decreases. Accordingly, the amounts of citrate and acetyl‐CoA increase, and the increased acetyl‐CoA inhibits autophagy/mitophagy. After that, synaptic plasticity is reduced by decreased autophagy/mitophagy, and as a result, AMI appears in middle‐aged flies.

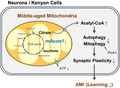

## 1 INTRODUCTION

Age‐related memory impairment (AMI) refers to a significant decrease in learning and memory ability with age (Gkikas et al., 2014). Approximately 40% of people over 60 years of age experience AMI. Notably, AMI results in decreased acumen to encode working memories of new events or facts (Klencklen et al., 2017). Moreover, AMI also can appear in middle age (Kwon et al., 2016). The known causes of AMI in humans are age‐related changes in the levels of cortisol, and estrogen (Jacobs et al., 1998; Lupien et al., 1998), cerebral vascular supply (Martin et al., 1991), oxidative stress (Lu et al., 2004), and a deficiency in the histone‐binding protein RbAp48 (Pavlopoulos et al., 2013).

AMI occurs in most animals, including nematodes, fruit flies, mice, and chimpanzees. In fruit flies, the AMI phenotype has been described as a decrease in learning (3‐min memory; Fresquet & Médioni, 1993), short‐term memory (<1 h; Yamazaki et al., 2014), middle‐term memory (3–7 h; Haddadi et al., 2014), or long‐term memory (>24 h; Tonoki & Davis, 2015). DC0 (cAMP‐dependent protein kinase) (Yamazaki et al., 2007), pyruvate decarboxylase (Yamazaki et al., 2014), and insulin‐like growth factor signaling molecules (Tanabe et al., 2017) in energy metabolism; antioxidant SOD1 (Haddadi et al., 2014) in oxidative stress; and TORC2 and UNC‐51 (Huang et al., 2013; Mochizuki et al., 2011) in autophagy have been reported as *Drosophila* AMI regulators. However, the mechanisms of AMI that emerge in middle‐aged individuals before the aging traits remain largely unknown.

There are two isoforms of aconitase: mitochondrial aconitase (m‐aconitase) and cytosolic aconitase (c‐aconitase). The human homolog of mitochondrial Acon1 (*mAcon1*; m‐aconitase in *Drosophila*) is aconitase 2 (*ACO2*). mAcon1 converts citrate to isocitrate in the tricarboxylic acid (TCA) cycle in the mitochondrial matrix (Beinert & Kennedy, 1993). When mAcon1 activity decreases, cellular respiration and ATP levels also decrease. These changes are associated with the dysfunction or senescence of cells. In addition, the iron–sulfur [4Fe‐4S] cluster of mAcon1 is involved in regulating iron homeostasis (Lauble et al., 1992) and interacts with PTEN‐induced kinase 1 (Pink1) (Esposito et al., 2013). In *Drosophila*, homozygous mutants of *mAcon1* are lethal. RNA interference (RNAi)‐mediated knockdown of *mAcon1* resulted in reduced locomotor activity, shortened lifespan, and increased cell death in the developing brain (Cheng et al., 2013). Thus, mAcon1 plays an essential role in mitochondrial activity, energy metabolism, and aging.

Neurons maintain proteostasis to fulfill high metabolic demands and manage various stresses (Yin et al., 2016). Autophagy is an essential mechanism to manage the homeostasis of proteins and organelles; decreased autophagy with aging contributes to brain aging (Aman et al., 2021; Rubinsztein et al., 2011). In autophagy, autophagy‐related proteins (ATGs) form autophagosomes and combine with lysosomes to degrade target organelles or proteins. Of them, ATG8 (*Drosophila* homolog of light chain 3, LC3) plays an important role in autophagosome maturation (lipidation), and p62 (ubiquitin‐binding scaffold protein) recognizes ubiquitinated proteins and interacts with ATG8, leading to elimination via autophagy (Pankiv et al., 2007). Therefore, p62 aggregates accumulate when autophagy is absent, and p62 level decreases when autophagy is induced.

Mitophagy is a selective form of autophagy that removes dysfunctional mitochondria to reorganize specific mitochondrial networks and to improve mitochondrial quality (Ashrafi & Schwarz, 2013). Mitophagy also eliminates superfluous healthy mitochondria in certain physiological conditions, such as the maturation of erythrocytes and removal of paternal mitochondria in fertilized oocytes (Sandoval et al., 2008). Pink1 and Park (E3 ubiquitin ligase) are necessary factors for mitophagy to occur (Lazarou et al., 2015). The suppression of *pink1* and *park* expression in *Drosophila* induces the accumulation of swollen and clumped mitochondria in dopamine neurons owing to the absence of proper mitophagy (Cornelissen et al., 2018). Interestingly, mAcon1 is a dominant suppressor of Pink1, and its expression levels are reduced in *pink1* mutants (Esposito et al., 2013). Therefore, mAcon1 likely regulates AMI through autophagy and mitophagy.

A recent report suggested that synaptic plasticity, rather than the loss of neurons, causes age‐related cognitive decline (Morrison & Baxter, 2012). Bruchpilot (BRP), a structural protein in the presynaptic active zone (AZ), regulates synaptic plasticity by controlling the release of synaptic vesicles into the synaptic cleft (Böhme et al., 2016). BRP content increases with age in *Drosophila* due to decreased autophagy (Gupta et al., 2016). This condition sets the upper limit of the operational range of AZ, limiting synaptic plasticity and contributing to impairment of memory formation. Interestingly, increased autophagy reduces brain BRP levels and improves AMI in *Drosophila* (Gupta et al., 2016). Hence, autophagy is closely related to synaptic plasticity, and autophagy induced by *mAcon1* expression is likely to regulate AMI.

Herein, we report that the metabolic regulator mAcon1 alters autophagic and mitophagic activities and induces further changes in synaptic plasticity to regulate AMI in middle‐aged flies.

## 2 RESULTS

### 2.1 AMI impairs learning in middle‐aged wild‐type fruit flies

**FIGURE 1:**
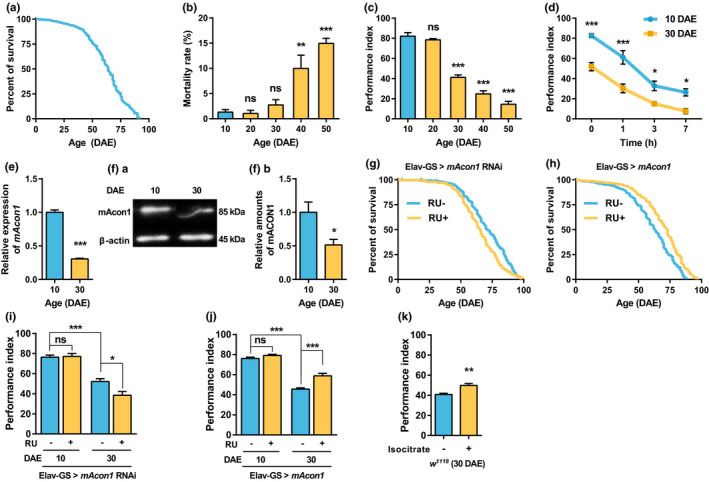
AMI appears in middle‐aged wild‐type flies, and the neuronal expression of *mAcon1* controls the lifespan and AMI. (a) Lifespan curve of *w^1118^*. The median lifespan (MLS) is 64 d, and the maximum lifespan (MaxLS) is 92 d (*n* = 712). (b) The mortality rate of *w^1118^* increases after 40 d after eclosion (DAE) (*F*_(4, 25)_ = 6.34, *p* < 0.01). (c) Compared to young flies, learning is significantly reduced in middle‐aged flies (*F*_(4, 35)_ = 125.2, *p* < 0.001). (d) Memory retention curve of *w^1118^*. The learning, and 1, 3, and 7 h memory of middle‐aged flies are significantly reduced, compared to the values from young flies (age effect [*F*_(1, 72)_ = 71.17, *p* < 0.001], time effect [*F*_(3, 72)_ = 76.62, *p* < 0.001], and interaction [*F*_(3, 72)_ = 1.62, *p* = 0.191]). (e and f) In *w^1118^* fly heads, the *mAcon1* mRNA and protein expression levels decrease significantly in middle‐aged flies, compared to young flies. (g and h) When *mAcon1* expression in neurons is reduced in adult stage, MLS decreases (g, RU‐: 71 d, *n* = 679; RU+: 65 d, *n* = 664; χ^2^ = 44.47, df = 1, *p* < 0.001). When *mAcon1* expression increases, MLS also increases (h, RU‐: 63 d, *n* = 694; RU+: 73 d, *n* = 700; χ^2^ = 74.52, df = 1, *p* < 0.001). (I and J) When *mAcon1* expression decreases in neurons in adulthood, AMI worsens (I; age effects [*F*_(1, 28)_ = 114.7, *p* < 0.001], RU effects [*F*_(1, 28)_ = 4.86, *p* < 0.05], and interaction [*F*_(1, 28)_ = 5.99, *p* < 0.05]). When *mAcon1* expression increases, AMI is alleviated (J, age effect [*F*_(1, 28)_ = 254.1, *p* < 0.0001], RU effect [*F*_(1, 28)_ = 25.87, *p* < 0.0001], and interaction [*F*_(1, 28)_ = 10.36, *p* < 0.01]). (K) Feeding *w^1118^* middle‐aged flies with isocitrate (50 µg/ml) for 30 d significantly improves learning (*n* = 8)

The lifespan curve reveals the level of progression of aging, wherein the premortality plateau is regarded as old age (Tamura et al., 2003). The lifespan of *w^1118^* is longer than that of Canton‐S‐related strains used in other AMI studies (Tanabe et al., 2017; Yamazaki et al., 2014). In this study, the median lifespan (MLS) of wild‐type *Drosophila* (*w^1118^*) combined with men and women was 64 days (d) (Figure 1a). This was much longer than that of Canton‐S‐related strains (*w^CS10^*, 33 d) (Paik et al., 2012). The mortality rate of Canton‐S increased 20 d after eclosion (DAE) (Tamura et al., 2003); however, this rate increased after 50 DAE for *w^1118^* (Figure 1b). Since *w^1118^* reached a premortality plateau at 56 d in men and 61 d in women, these flies can be considered middle aged before 50 DAE. Expression of antioxidant genes, such as *cat* and *sod1*, is decreased in old flies compared to young flies (Haddadi et al., 2014). However, *cat* and *sod1* mRNA expression levels in *w^1118^* did not decrease until 30 DAE (Figure S1e,f). Because no evident aging phenotypes in mortality and expression of antioxidant genes appeared at 30 DAE, we considered *w^1118^* flies at 10 DAE as young flies and 30 DAE as middle‐aged flies.

Using the aversive olfactory learning/memory paradigm, we examined the AMI phenotype in learning at 10‐d intervals from 10 to 50 DAE in *w^1118^*. Learning (3‐min memory) ability decreased significantly after 30 DAE (Figure 1c). There was no difference in the odor‐sensing ability to benzaldehyde (BEN) or 3‐octanol (OCT) and reactivity to electric shock between *w^1118^* at 10 and *w^1118^* at 30 DAE (Figure S1a‐c). Anti‐geotactic locomotor activity was slightly reduced at 30 DAE than at 10 DAE (Figure S1dd). However, as no difference was observed in the avoidance tests at both 10 and 30 DAE, reduced anti‐geotactic locomotor activity at 30 DAE did not affect the learning tests.

After learning, memory retention ability decreased significantly over time (Figure 1d). The performance index (PI) of 10 DAE young flies was significantly higher than that of 30 DAE middle‐aged flies. Notably, the slope of the memory retention curve of the young flies was similar to that of the middle‐aged flies. Therefore, the AMI appearing in the middle‐aged *w^1118^* flies was not a memory impairment but rather a learning (3‐min memory) impairment.

### 2.2 *mAcon1* regulates longevity

In the heads of *w^1118^* flies, the expression levels of *mAcon1* mRNA (Figure 1e) and protein (Figure 1fa‐fb) significantly decreased as aging progressed. *mAcon1* transcript and protein levels in isolated heads of the *actin*‐GeneSwitch [GS]‐Gal4 > *mAcon1* RNAi (RU486‐positive [RU+]) were reduced by almost 50% and 60% compared to controls (RU‐), respectively (Figure S3a,c,d). The *mAcon1* overexpression efficiency increased 5.6‐fold at the mRNA level and 1.6‐fold at the protein level, compared to that in control flies (Figure S3b,c,e). To determine whether *mAcon1* expression affects lifespan, we measured the lifespan of genetically modified adult flies in which *mAcon1* expression was modulated by an inducible pan‐neuronal Gal4 driver and its inducer RU continuously after eclosion. When the neuronal expression of *mAcon1* was suppressed, MLS significantly decreased in adult flies (8.45%, Figure 1g). When *mAcon1* expression was upregulated, MLS significantly increased (15.87%, Figure 1h). Thus, *mAcon1* is an aging‐ and longevity‐regulating gene.

### 2.3 Neuronal expression of *mAcon1* regulates AMI in *w^1118^* flies

In young flies, the neuronal increase or decrease in *mAcon1* expression levels did not affect learning (Figure 1i‐j). In middle‐aged flies, downregulating *mAcon1* expression in neurons led to a significant decrease in learning (Figure 1i). Conversely, neuronal overexpression of *mAcon1* increased learning (Figure 1j). Therefore, the neuronal control of *mAcon1* expression after eclosion regulated AMI in middle‐aged flies.

### 2.4 Feeding isocitrate, a metabolic product of mAcon1, improves AMI

**FIGURE 2:**
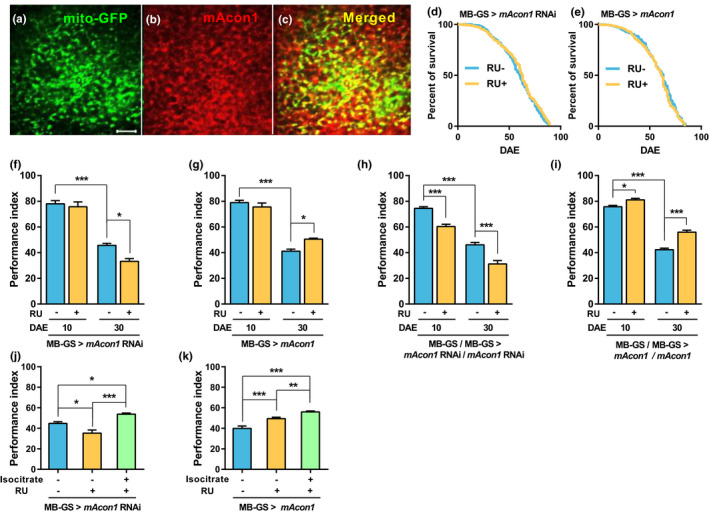
Modulation of *mAcon1* expression in Kenyon cells (KCs) regulates AMI. (a–c) Mitochondria of KCs labeled with (a, MB‐GS >mito‐GFP, scale bar: 10 µm) GFP and *mAcon1* labeled with (b) *mAcon1*‐specific antibodies colocalized (c, 74.95 ± 3.91%, *n* = 5). (d and e) Survival curve patterns and MLS are not changed by reducing (d, RU‐: 60 d, *n* = 703; RU+: 63 d, *n* = 670; χ^2^ = 2.709, df = 1, *p* = 0.1) or increasing (e, RU‐: 63 d, *n* = 700; RU+: 61 d, *n* = 698; χ^2^ = 2.50, df = 1, *p* = 0.114) *mAcon1* expression in the KCs. (f and g) Reducing *mAcon1* expression in KCs during the adult period of middle‐aged flies significantly reduces learning (f, age effect [*F*_(1, 28)_ = 205.1, *p* < 0.001], RU effect [*F*_(1, 28)_ = 7.916, *p* < 0.01], and interaction [*F*_(1, 28)_ = 3.81, *p* = 0.061]). Increasing *mAcon1* expression improves learning (g, age effect [*F*_(1, 28)_ = 257.4, *p* < 0.01]). (h) Reinforcing the intensity of *mAcon1* knockdown in KCs of the young flies decreased learning (age effect [*F*_(1, 28)_ = 226.5, *p* < 0.001], and RU effect [*F*_(1, 28)_ = 57.64, *p* < 0.001]). (I) Reinforcing the intensity of *mAcon1* overexpression in KCs of the young flies increases learning (age effect [*F*_(1, 28)_ = 578.8, *p* < 0.001], RU effect [*F*_(1, 28)_ = 60.53, *p* < 0.001], and interaction [*F*_(1, 28)_ = 11.66, *p* < 0.01]). (j and k) Feeding isocitrate (50 µg/ml) for 30 d improves AMI, regardless of *mAcon1* expression levels in the KCs of middle‐aged flies (j, *F*_(2,29)_ = 19.46, *p* < 0.001; k, *F*_(2,29)_ = 28.2, *p* < 0.001)

If mAcon1 is an essential factor in AMI, it is plausible that isocitrate, a catalytic product of mAcon1, may also affect AMI. To assess this, we fed the middle‐aged *w^1118^* with various concentrations of isocitrate and measured learning afterward. Learning was significantly improved when the middle‐aged flies were fed 50 µg/ml isocitrate (Figure 1k and Figure S2a). However, there was no improvement in learning at the same concentration in young flies (Figure S2b). Moreover, feeding isocitrate improved AMI, even when *mAcon1* expression levels decreased in the mushroom body (MB) (Figure 2j and Figure S2c). Feeding isocitrate for 7 d to flies with overexpressed *mAcon1* in the MB did not improve AMI (Figure S2d). However, feeding it for 30 d further improved AMI (Figure 2k). Hence, AMI could be improved through the supplementation of isocitrate.

### 2.5 Changes in *mAcon1* expression in the MB do not affect lifespan

*The mAcon1* encodes one of the TCA cycle enzymes in the mitochondria. The enzyme is present in all neural cells (Beinert & Kennedy, 1993). Immunohistochemistry (IHC) using *mAcon1*‐specific antibody demonstrated that *mAcon1* was expressed in the whole brain (Figure S3d‐f). The protein colocalized with the mitochondria of the Kenyon cells (KCs) (Figure 2a‐c); KCs construct MB neuropils and function in olfactory learning and memory in fruit flies (Aso et al., 2014). Unlike the neuronal effects of *mAcon1* on longevity (Figure 1g‐h), changes in *mAcon1* expression in the MB during adulthood did not affect MLS or the patterns of survival curves (Figure 2d,e).

### 2.6 *mAcon1* expression levels in the MB regulate AMI

To determine whether *mAcon1* expression levels in the KCs are related to AMI, we evaluated learning of a *mAcon1* mutant and flies with modulated *mAcon1* expression using MB247‐Gal4. Young *mAcon1* mutant flies (*mAcon1^mb09176^*) displayed significantly reduced learning (Figure S4a). Learning was not affected in Gal4 heterozygotes (MB247‐Gal4/+ > RNAi/+ or > UAS/+). In contrast, Gal4 homozygotes (MB247‐Gal4/MB247‐Gal4 > RNAi/+ or > UAS/+) demonstrated decreased learning in young flies (Figure S4b). In middle‐aged flies, learning was reduced even in Gal4 heterozygotes (Figure S4c). Next, we measured AMI after modulating *mAcon1* mRNA levels in the KCs throughout the adult stage using inducible Gal4 (MB‐GS‐Gal4). In the case of heterozygotes, the increase or decrease in *mAcon1* expression in the KCs of young flies did not affect learning. However, in middle‐aged flies, the decrease in *mAcon1* expression significantly worsened AMI and increased *mAcon1* expression improved learning (Figure 2f,g). When we increased the working intensity by constructing the homozygous Gal4/UAS system (MB‐GS‐Gal4/MB‐GS‐Gal4 > RNAi/RNAi or >UAS/UAS), the learning ability was regulated according to the *mAcon1* expression levels in the KCs, even in young flies (Figure 2h‐i). To determine whether short‐term control of *mAcon1* could affect learning, we limited RU feeding to 7 d. There was no significant difference in learning between heterozygotes (Figure S5a,b). Short‐term feeding of RU significantly decreased learning in the homozygous young flies but not in middle‐aged flies (Figure S5c,d). These results suggest that both the expression levels and the available duration of *mAcon1* were essential for learning, and *mAcon1* in KCs could affect the learning of the young and middle‐aged flies.

### 2.7 Aging and MB *mAcon1* expression levels control ATP production

**FIGURE 3:**
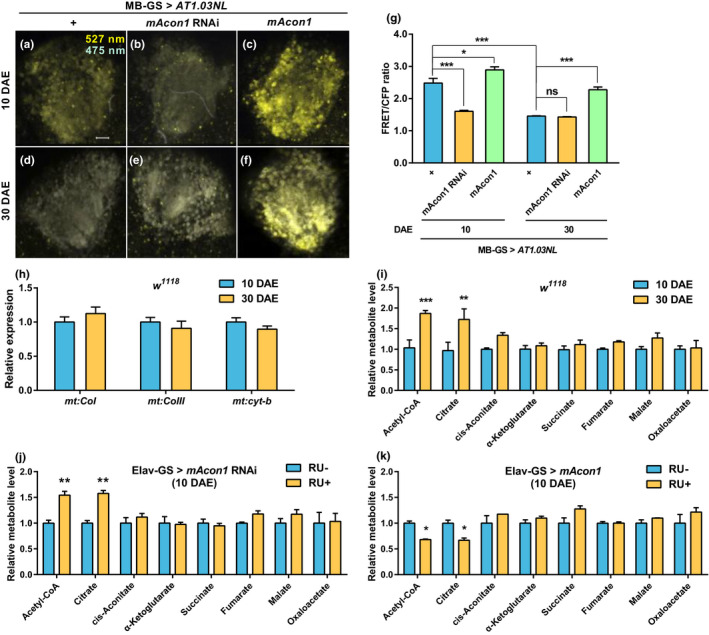
Expression levels of *mAcon1* affect citrate, acetyl‐CoA, and ATP contents. (a–g) ATP in KCs of young or middle‐aged flies estimated by normalizing the FRET signal of ATP reporter (MB‐GS >AT1.03NL) to CFP (ATP unbinding form) (a–f, scale bar: 10 µm). ATP content decreases significantly in middle‐aged flies compared to that in KCs of young flies. *mAcon1* overexpression significantly increases ATP production (g, age effect [*F*_(1, 22)_ = 80.52, *p* < 0.001], genotype effect [*F*_(2, 22)_ = 89.26, *p* < 0.001], and interaction [*F*_(2, 22)_ = 12.40, *p* < 0.001]). (h) No significant difference in the amount of mitochondrial DNA in the heads of the *w^1118^* young and middle‐aged flies. (i) Acetyl‐CoA and citrate levels are significantly increased in the heads of middle‐aged flies compared to that in *w^1118^* young flies (age [*F*_(1,32)_ = 29.72, *p* < 0.001] and metabolite effects [*F*_(7,32)_ = 3.34, *p* < 0.01]). (j and k) Reducing *mAcon1* expression in neurons during the adult stage of young flies significantly increases the levels of acetyl‐CoA and citrate in the head (j, age [*F*_(1,32)_ = 16.58, *p* < 0.001] and metabolite effects [*F*_(7,32)_ = 3.25, *p* < 0.05]), whereas increasing *mAcon1* expression significantly decreases acetyl‐CoA and citrate levels (k, metabolite effect [*F*_(7,32)_ = 4.66, *p* < 0.01])

As the expression of mAcon1 and other TCA cycle enzymes decreases with aging, the amount of ATP produced should also decrease. To verify this, we estimated the change in ATP levels in KCs using AT1.03NL, an ATP reporter (Tsuyama et al., 2013). As aging progressed, the amount of ATP in the KCs decreased significantly in middle‐aged flies compared to young flies (Figure 3a,d,g). Next, we investigated whether the regulation of *mAcon1* expression affects ATP production. In young flies, the amount of ATP significantly decreased (Figure 3b,g) or increased (Figure 3c,g), depending on the expression levels of *mAcon1*. Similarly, when *mAcon1* expression in the KCs increased, ATP significantly increased in the middle‐aged flies (Figure 3f‐g). However, the reduction in *mAcon1* expression did not decrease ATP levels in the middle‐aged flies (Figure 3e,g). This discrepancy might have reflected the detection limit of AT1.03NL.

### 2.8 Aging and *mAcon1* expression do not affect mitochondrial DNA content

To determine whether the changes in the amount of ATP are related to the copy number of mitochondria in the KCs, we estimated the mitochondrial copy number by quantifying mitochondrial genes. The genes included mitochondrial cytochrome c oxidase subunit I (*mt*:*CoI*), mitochondrial cytochrome c oxidase subunit III (*mt*:*CoIII*), and mitochondrial cytochrome b (*mt*:*Cyt*‐*b*). We observed no difference in the number of these mitochondrial genes in the heads of young and middle‐aged *w^1118^* flies (Figure 3i). Next, after labeling mitochondria with the fluorescent protein mt‐Keima, *mAcon1* expression was regulated in the KCs. The KCs expressing the mt‐Keima signal were collected using fluorescence‐activated cell sorting (FACS), and the mitochondrial genes were quantified. Aging and *mAcon1* expression levels caused no significant difference in mitochondrial DNA copy number between the control and experimental groups where *mAcon1* expression levels were modulated (Figure S7a‐c).

### 2.9 Aging and neuronal *mAcon1* expressions are associated with acetyl‐CoA and citrate levels

Because *mAcon1* expression decreased with aging, we speculated that reducing TCA cycle metabolites plausibly decrease ATP production. To verify this, we quantified these metabolites in fly heads using liquid chromatography‐mass spectrometry (LC‐MS). Only acetyl‐CoA and citrate concentrations were significantly enriched in the head of middle‐aged flies and not in young flies (Figure 3i). Levels of other TCA cycle metabolites were not different between the young and middle‐aged flies. Interestingly, neuronal suppression of *mAcon1* in young flies significantly increased the levels of acetyl‐CoA and citrate (Figure 3j). These results were similar to the metabolite patterns observed in the middle‐aged *w^1118^* flies (Figure 3i). Conversely, increasing *mAcon1* expression significantly reduced acetyl‐CoA and citrate levels (Figure 3k) in young flies. Thus, the expression level of *mAcon1* may be essential for maintaining acetyl‐CoA, citrate, and ATP levels in middle‐aged flies.

### 2.10 *mAcon1* expression in the MB regulates mitophagy

**FIGURE 4:**
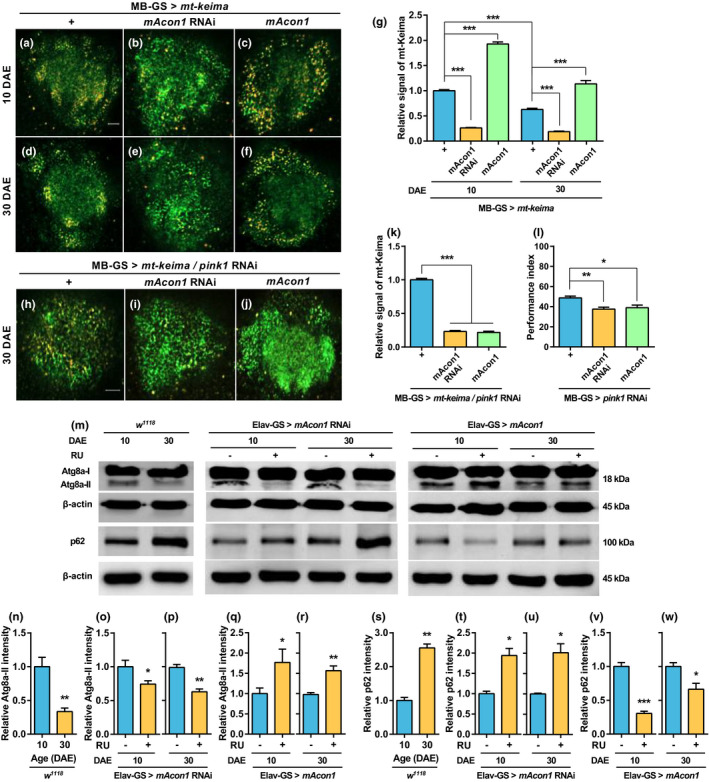
Aging and *mAcon1* regulates autophagy and mitophagy. (a–f) mt‐Keima emission signals from *Drosophila* (MB‐GS >mt‐Keima) expressing the mitophagy reporter mt‐Keima protein in KCs (scale bar: 10 µm). (g) Yellow‐orange mt‐Keima signal (low pH) decreases significantly with aging but significantly increases as *mAcon1* expression increases (age effect [*F*_(1, 56)_ = 207.9, *p* < 0.001], genotype effect [*F*_(2, 56)_ = 709.6, *p* < 0.001], and interaction [*F*_(2, 56)_ = 53.94, *p* < 0.001]). (h–j) In the KCs of middle‐aged flies, mitophagy induced by increased *mAcon1* expression disappears when *pink1* expression is suppressed (h, scale bar: 10 µm; k, *F*_(2, 27)_ = 662.5, *p* < 0.001). (l) When *pink1* is knocked down in middle‐aged flies, no improvement in AMI is observed because of *mAcon1* overexpression (*F*_(2, 21)_ = 8.15, *p* < 0.01). (m) Immunostaining of protein expression levels of ATG8a‐II and p62 reveals changes according to age and expression level of mAcon1 in the heads of *w^1118^* flies and neuronal modulation of mAcon1. (n‐w) Expression levels of Atg8a‐II and p62 proteins according to aging and expression level of mAcon1 (*n* = 4–6; using *t*‐test). (n and s) Expression of Atg8a‐II (n) and p62 (s) in the head of *w^1118^* flies significantly reduces in middle‐age. (o–r and t‐w) Regulation of Atg8a‐II and p62 levels by the neuronal expression of *mAcon1* in young (o–r) and middle‐aged (t–w) flies. The results are normalized to β‐actin levels

The findings that *mAcon1* controls ATP production without changing the total mitochondrial DNA content in KCs prompted an experiment to verify the relationship between changes in ATP production and mitochondrial quality control via mitophagy (Ashrafi & Schwarz, 2013). The mt‐Keima protein was expressed in KCs to visualize mitophagy *in vivo* (Sun et al., 2015). The yellow‐orange signal of mt‐Keima decreased with aging (Figure 4a,d,g). Moreover, when the expression level of *mAcon1* decreased, the mt‐Keima signal was significantly reduced in both young and middle‐aged flies (Figure 4b,e,g). Similarly, when the expression level of *mAcon1* increased, the mt‐Keima signal significantly increased in young and middle‐aged flies (Figure 4c,f,g).

*The mAcon1* expression was modulated in KCs labeled with mt‐Keima to investigate the correlation between *mAcon1* and various mitophagy regulators (Gkikas et al., 2018). KCs emitting the mt‐Keima signal were collected using FACS. The mRNA levels of mitophagy regulatory genes, such as AMP‐activated protein kinase alpha subunit (*AMPKα*), target of rapamycin (*tor*), and the sirtuin family members, were measured. Only *AMPKα* mRNA levels changed according to *mAcon1* expression (Figure S8a‐g).

### 2.11 *The pink1* is required for mitophagy induction and AMI improvement

*The pink1* is necessary for mitophagy induction (Lazarou et al., 2015), and *mAcon1* is a dominant suppressor of *pink1* (Esposito et al., 2013). Therefore, we investigated whether mitophagy induction by *mAcon1* was related to *pink1*. The mRNA expression level of *pink1* in the heads of *w^1118^* flies decreased with age (Figure S9a). When the expression of *pink1* in *Drosophila* heads was modulated using *actin*‐GS‐Gal4, the expression level of *mAcon1* did not change (Figure S9b,c). Conversely, even when *mAcon1* expression was manipulated under the same conditions, the expression levels of *pink1* remained unchanged (Figure S9d,e). Mitophagy significantly decreased when the expression of *pink1* was suppressed after controlling *mAcon1* expression in the KCs of middle‐aged flies (Figure 4h‐k). Furthermore, *mAcon1* expression levels showed no significant deterioration or improvement effects on AMI in middle‐aged flies when *pink1* expression was repressed (Figure 4l). These results imply that the *pink1* pathway is required to induce mitophagy and improve AMI through *mAcon1*.

### 2.12 Aging and *mAcon1* modulate autophagy

Next, the expression levels of ATG8a and p62 proteins were quantified using Western blotting to determine whether aging and the regulation of *mAcon1* expression affect autophagy. Regarding autophagy activity, the level of ATG8a‐II, a lipidated form of ATG8a, was significantly lower in middle‐aged flies than in young flies (Figure 4m,n). When *mAcon1* expression decreased in neurons, the amount of ATG8a‐II also decreased in both young and middle‐aged flies (Figure 4m,o,p). Similarly, when mAcon1 expression increased in neurons, the amount of ATG8a‐II also increased in both aged flies (Figure 4m,q,r). To estimate autophagic flux, we also measured the expression level of the ubiquitin‐binding scaffold protein p62 (*Drosophila* homolog, Ref(2)P). Expression levels of p62 significantly increased in middle‐aged flies compared to young flies (Figure 4m,s). When mAcon1 expression decreased in neurons, p62 also increased in young and middle‐aged flies (Figure 4m,t,u). When mAcon1 expression increased in neurons, the amount of p62 decreased in both aged flies (Figure 4m,v,w). Thus, neuronal *mAcon1* expression can regulate the production and degradation of autophagosome during autophagy. Considering the mitophagy results (Figure 4a‐g), it could be concluded that *mAcon1* regulates both autophagy and mitophagy in the *Drosophila* brain.

### 2.13 Neuronal *mAcon1* expression regulates BRP protein levels in whole brain

**FIGURE 5:**
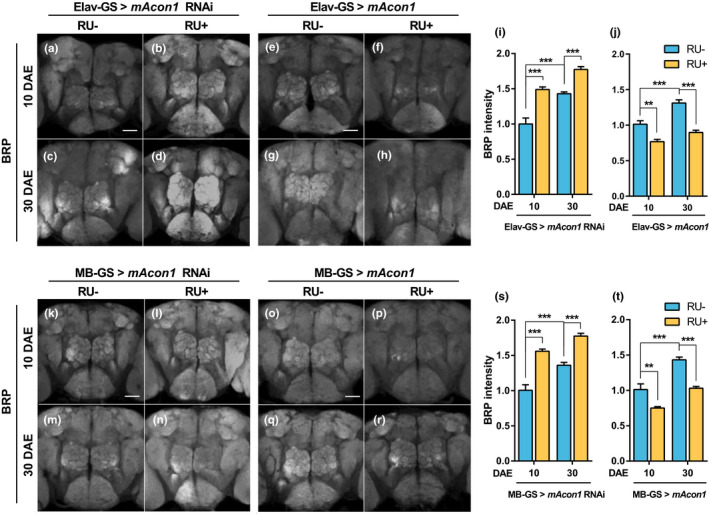
Aging and *mAcon1* regulate the expression of the presynaptic structural protein BRP in the whole brain. (a–h) Neuronal expression levels of *mAcon1* were controlled. BRP expression visualized using IHC (a and e, scale bar: 10 µm). (i–j) BRP expression increases significantly with aging and is inversely proportional to *mAcon1* expression levels (I, age [*F*_(1, 20)_ = 58.63, *p* < 0.001] and genotype effects [*F*_(1, 20)_ = 81.78, *p* < 0.001]; j, age effect [*F*_(1, 20)_ = 28.76, *p* < 0.001], genotype effect [*F*_(1, 20)_ = 68.12, *p* < 0.001], and interaction [*F*_(1, 20)_ = 4.53, *p* < 0.05]). (k–o) *mAcon1* expression levels were controlled in the MB KCs. Visualization of brain BRP distribution (k and o, scale bar: 10 µm). (s–t) Although *mAcon1* expression is controlled only in KCs, changes in BRP signals appear in the whole brain. The pattern is inversely proportional to *mAcon1* expression levels shown in panels i–j. (s, age [*F*_(1, 20)_ = 31.96, *p* < 0.001] and genotype effects [*F*_(1, 20)_ = 91.61, *p* < 0.001]; t, age [*F*_(1, 20)_ = 54.08, *p* < 0.001] and genotype effects [*F*_(1, 20)_ = 48.32, *p* < 0.001])

Reduction in autophagic activity in the *Drosophila* brain results in a learning decline because of decreased synaptic plasticity (Bhukel et al., 2017). Moreover, the activity of synaptic plasticity can be estimated by the amount of the presynaptic structural protein BRP (Bhukel et al., 2019). Thus, we measured BRP levels to understand the possible role of *mAcon1* in synaptic plasticity after manipulating *mAcon1* expression in young and middle‐aged flies. As aging progressed, BRP expression increased significantly in the brain (Figure 5a,c,e,g,I,h). When *mAcon1* expression decreased in neurons, the BRP expression levels increased significantly in young and middle‐aged flies (Figure 5b,d,i). Conversely, an increase in *mAcon1* expression significantly decreased the expression of BRP protein in both the young and middle‐aged flies (Figure 5f,h,j).

### 2.14 *The mAcon1* expression in the MB also regulates BRP protein levels in whole brain

When autophagy is controlled only in the MB, a synaptic phenotype appears in the whole fly brain in a non‐cell‐autonomous manner (Bhukel et al., 2019). We controlled *mAcon1* expression levels in the MB and measured BRP expression in the whole brain to determine the non‐cell‐autonomous effects of *mAcon1*. Flies in which *mAcon1* expression decreased in the KCs displayed significantly increased BRP expression with age (Figure 5k,m,o,q,s,t). Moreover, as in the case where *mAcon1* expression decreased in neurons, the reduction in *mAcon1* expression only in the MB significantly increased BRP levels in the whole brain (Figure 5l,n,s). Furthermore, when *mAcon1* expression increased, BRP levels decreased (Figure 5p,r,t). These results indicate that *mAcon1* expression in all neurons or only the MB can regulate a non‐cell‐autonomous effect on the synaptic plasticity of the whole brain.

## 3 DISCUSSION

AMI is affected by energy metabolism, autophagy/mitophagy, and synaptic plasticity. The results of this study demonstrate that AMI, defined as a learning impairment in middle‐aged flies, is regulated by mAcon1, a mitochondrial TCA cycle enzyme. In addition to the findings by Gupta et al. (2013) who proposed that lowering the neuronal autophagic clearance by aging is related to AMI, we reveal that mAcon1 regulates autophagy/mitophagy and further neural plasticity to control AMI.

We observed an increase in acetyl‐CoA and citrate levels in fly heads with aging and upon *mAcon1* suppression (Figure 3i‐j). When the expression of *mAcon1* increased, the contents of citrate and acetyl‐CoA decreased in the fly heads (Figure 3k). The increased citrate moves into the cytoplasm via citrate carriers and is converted to acetyl‐CoA and oxaloacetate by ATP‐citrate lyase. Therefore, inhibition of *mAcon1* expression increases the amount of cytosolic acetyl‐CoA (Wellen et al., 2009). Increased cytosolic acetyl‐CoA decreases autophagy. Conversely, when acetyl‐CoA decreased, autophagy increased (Figure 4a‐g,m‐w). Acetyl‐CoA acts as a cellular energy sensor to regulate autophagy in the nucleus and cytoplasm through three mechanisms. First, increased acetyl‐CoA in the nucleus promotes histone acetylation through the action of lysine acetyltransferases (KATs) to inhibit *ATG* gene expression and thereby inhibits autophagy (Eisenberg et al., 2014). Moreover, p300/CBP inhibits autophagy by acetylation of FOXOs in the nucleus (Bánréti et al., 2013). Second, when acetyl‐CoA level in the cytoplasm increases, the acetylation of several proteins, including autophagy mediator proteins, through p300/CBP increases, and autophagy is inhibited (Mariño et al., 2014; McEwan & Dikic, 2011). Several enzyme groups, including GNAT, p300/CBP, Rtt109, and MYST, acetylate cellular proteins (Dancy & Cole, 2015). However, the enzymes that mediate cellular acetyl‐CoA levels to induce autophagy are unknown. The third mechanism involves the histone deacetylase 6 (HDAC6) pathway through which acetyl‐CoA also affects autophagy. When acetyl‐CoA levels increase, deacetylation of α‐tubulin by HDAC6 decreases, mitochondrial transport through microtubules is improved, and subsequently, mitophagy is reduced (Lee et al., 2010). HDAC6 promotes autophagosome–lysosomal fusion by deacetylation of cortactin to activate F‐actin remodeling (Lee et al., 2010). The expression of ecdysone‐induced protein 74EF *(Eip74EF)*, a target gene of *nejire* (*Drosophila* homolog of p300/CBP) (Bodai et al., 2012), was significantly increased in *mAcon1*‐knockdown flies and decreased in *mAcon1*‐overexpressing flies (Figure S10). These findings imply that cellular acetyl‐CoA should regulate the activity of the nuclear p300/CBP. How the changes in cellular acetyl‐CoA levels by mAcon1 regulate autophagy needs to be studied.

The ongoing structural reorganization of neuronal circuits through neuronal plasticity is essential for acquiring knowledge and consolidation of memory (Fleming & Rubinsztein, 2020). In the presynaptic AZ, scaffold proteins, such as BRP, control synaptic plasticity by regulating synaptic vesicle (SV) release factors like unc‐13 (Böhme et al., 2016). Autophagy plays an important role in synaptic plasticity (Kulkarni & Maday, 2018). The size of AZ increases as aging progresses or autophagy decreases (Gupta et al., 2016). The operational range of synapse reaches the upper limit (metaplasticity), the synapse is gradually strengthened, and plasticity decreases; eventually learning ability is reduced (Bhukel et al., 2017). Autophagy and BRP expression are closely related. For example, spermidine inhibits AMI by decreasing the size of presynaptic AZ and SV release, which increases with aging (Gupta et al., 2016). Additionally, knockdown of *atg5* and *atg9* among autophagy machinery genes increases BRP level and decreases memory capacity in presynaptic AZ (Bhukel et al., 2019). In our study, although we could not measure the amount of BRP after direct autophagy control, autophagy/mitophagy activity in the head and KCs decreased with aging (Figure 4), and BRP level in the brain increased (Figure 5). Conversely, when *mAcon1* expression increased, autophagy activity increased, and the amount of BRP decreased (Figures 4 and 5). These results suggest that autophagy induced by mAcon1 promotes neuronal plasticity through the micro‐processes mentioned above and further regulates age‐related memory.

The mAcon1 transcript and protein levels decreased during middle age in *Drosophila* (Figure 1e,f). It is important to elucidate the mechanism for the decrease in mAcon1 with aging to determine the fundamental cause of AMI. As aging progresses, the expression of genes encoding some TCA cycle enzymes was significantly reduced (Figure S6). These enzymes included citrate synthase (*kdn*), isocitrate dehydrogenase (*idh3a*), succinate dehydrogenase (*SdhA*), and oxoglutarate dehydrogenase (*Nc73EF*). In addition, some mitochondria‐related proteins and mAcon1 change (Manczak et al., 2005). The function of the mitochondrial oxidative phosphorylation complex and the expression of related genes also reduce with age in humans (Emelyanova et al., 2018). Recently, it was reported that when oxidative stress increased following *SOD* mutation, the mRNA and protein levels of aconitase decreased in *Salmonella enterica*, with increased expression of proteins involved in oxidative stress response and DNA damage repair (Noster et al., 2019). Therefore, the increase in oxidative stress due to aging may be one of the causes of decreasing aconitase mRNA and protein expression in *Drosophila*.

Since mAcon1 is one of the TCA cycle enzymes, its deficiency can cause various problems. In particular, inhibited *mAcon1* expression in all cells at the developmental stage affects locomotor activity, cell death, and lifespan (Cheng et al., 2013). To avoid the widespread effect of mAcon1, we used the inducible Gal4 system only for 10 or 30 DAE. Expression sites were also limited to neurons and MB KCs, and feeding 150 µM of RU does not affect the lifespan (Tricoire et al., 2009). Experimentally, the expression of *mAcon1* using the MB‐GS‐Gal4 driver did not change the *Drosophila* lifespan (Figure 2d,e). However, even under the same conditions, flies showed a significant difference in learning ability without any difference in sensory ability (Figures S1a,c and S2f‐k). Thus, we could avoid the adverse effects of mAcon1 in this study. In humans, patients with mAcon1 deficiency have cardiac palpations and various myopathies (Mochel & Haller, 1993). These symptoms appear to be related to the loss of control of iron–sulfur homeostasis by mAcon1. Although we mainly studied the metabolic effect of mAcon1, it would be interesting to study the function of mAcon1 in iron–sulfur homeostasis.

Well‐known lifespan signaling pathways, such as insulin‐like signaling, mitochondrial stress, and hypoxic response, operate through non‐cell‐autonomous signaling mechanisms (Miller et al., 2020). A neuron‐specific knockdown of *mAcon1* also extended the lifespan of *Drosophila* (Figure 1g). When aging progresses, the activity of mAcon1 in the thoracic flight muscles of *Drosophila* decreases (Yan et al., 1997). Feeding citrate increases the lifespan of *Drosophila* (Patel et al., 2016). Thus, the expression effect of mAcon1 may appear through acetyl‐CoA or citrate, even in peripheral tissues. In addition, since neuronal senescence can be promoted when autophagy is reduced (Kang et al., 2011; Moreno‐Blas et al., 2019), decreased expression of *mAcon1* in neurons reduces autophagy to promote senescence. These senescent neurons may induce peripheral tissue aging via the senescence‐associated secretory phenotype. Thus, the effect of neuronal *mAcon1* expression may be transmitted to the peripheral tissues that consequently affect the lifespan.

Using the mt‐Keima protein, we observed that local induction of *mAcon1* expression in the KCs modulated mitophagy (Figure 4f‐l). Furthermore, the expression of ATG8a‐II, an autophagosome‐forming protein, was regulated by *mAcon1* expression (Figure 4a‐e). In our IHC experiments, we labeled mitochondria with green fluorescent protein (GFP) and ATG8a using red fluorescent protein. Less than 6% of the signals were colocalized, and colocalization of morphologically intact mitochondria with ATG8a signal clumps was not observed (Figure S11a,b). It is possible that the mitochondria could be degraded rapidly within the autophagosome and that the GFP signal could be underestimated because GFP is not as stable as the mt‐Keima protein, especially in acidic lysosomes. Nevertheless, these results imply that *mAcon1* induces both selective and non‐selective autophagy.

We selected *mAcon1* from the screening studies to identify novel genes controlling longevity by measuring the lifespan of EP lines with a long‐lived *w^1118^* genetic background (Paik et al., 2012) and performed DNA microarray analysis with short‐lived cantonized *w^1118^* (*w^CS10^*). *mAcon1* expression decreases with age in humans (Emelyanova et al., 2018); the amount of cytosolic acetyl‐CoA increases in human U2OS cells, and autophagy decreases (Mariño et al., 2014). In mice, increased autophagy improves synaptic plasticity and suppresses age‐related memory decline (Glatigny et al., 2019). Thus, decreased expression of *mAcon1* with aging may occur in other *Drosophila* strains and other species. Confirming mAcon1 functions in autophagy and synaptic plasticity in other species would be exciting.

In conclusion, we found that AMI is a learning (3‐min memory) impairment in middle‐aged flies using wild‐type *Drosophila* (*w^1118^*) and demonstrated that mAcon1, a TCA enzyme, is a novel regulator of aging and AMI. The findings also reveal the interconnection of energy metabolism, autophagy, mitophagy, and synaptic plasticity and their involvements in AMI regulation through mAcon1. It would be interesting to determine the roles of mAcon1 in neurodegenerative and pathological conditions.

## 4 EXPERIMENTAL PROCEDURES

### 4.1 Fly stocks and culture

Fly stocks were incubated at 25°C, 50% relative humidity (RH), and a 12:12 h light‐dark cycle. *Drosophila* food was prepared using a cornmeal–sugar–glucose–yeast recipe. Both sexes were used in all the experiments. In knockdown, and overexpression studies using the GS Gal4 system, flies were transferred to medium containing RU after the F1 generation became adults. RU was dissolved in ethanol and mixed with fly food to a final concentration of 150 µM (Paik et al., 2012). During aging, the medium was replaced with a fresh medium every 2–3 d. All lines used in this study were outcrossed to *w^1118^* for at least 8 generations. *w^1118^*, *mAcon1^mb09176^*, *mAcon1*‐RNAi, UAS‐*mAcon1*, UAS‐mCD8‐GFP, UAS‐mito‐GFP, and *pink1*‐RNAi flies were obtained from the Bloomington *Drosophila* Stock Center (Bloomington, IN, USA); UAS‐AT1.03NL flies from the Kyoto Stock Center (Kyoto, Japan); and UAS‐mt‐Keima flies from Korea *Drosophila* Resource Center (Gwangju, South Korea). MB‐GS‐Gal4 and elav‐GS‐Gal4 flies were provided by Dr. M. Saitoe (Tokyo Metropolitan Institute of Medical Sciences) and Dr. M. Tatar (Brown University), respectively.

### 4.2 Aversive olfactory conditioning in flies

Standard single‐cycle aversive olfactory conditioning was performed as previously described with slight modifications (Yamazaki et al., 2007). In a dark room (dim‐red light, 25°C, 50% RH), 70–100 flies were placed in an electric shock vial for 90 s. Then, the flies were exposed to octanol (OCT) diluted 1:1000 with mineral oil as a conditioned stimulus (CS+) and a 60 V electric shock for 1.5 s as an unconditioned stimulus (US) 12 times for 1 min concurrently. After 30 s of rest, CS‐ flies were exposed to benzaldehyde (BEN) diluted 1:1000 in mineral oil for 1 min. After another 30 s of rest, the learning‐conditioned fruit flies were made to choose OCT or BEN. The outcome (PI) was calculated as (CS− − CS+) × 100 / (CS− + CS+). To exclude the directional bias of *Drosophila*, the average of each PI value obtained after testing with the positions of CS+/US and CS−inverted was used as a single measurement. Tests were conducted immediately after the training to measure learning. Tests were performed at 1, 3, and 7 h after training to draw the memory retention curve.

### 4.3 Reverse transcription‐PCR (RT‐PCR)

Total RNA was extracted from samples using RNAiso (TaKaRa Bio, Shiga, Japan), and its concentration was measured using a NanoDrop spectrophotometer (Thermo Fisher Scientific, Waltham, MA, USA); 1 µg of RNA was reverse‐transcribed to cDNA using a TP600 PCR Thermal Cycler Dice (TaKaRa Bio). The generated cDNA was stored at −80°C. RT‐PCR was performed using Power SYBR^®^ Green PCR Master Mix (Applied Biosystems), according to the instructions of the manufacturer, and Applied Biosystems™ 7500 Real‐Time thermal cycler. The primer sequences used in this study are shown in Table S2. The Ct values of all experimental groups were normalized to the Ct value of *rp49* and subtracted from that of the control group. The result was −ΔCt, and the fold change was calculated as 2^−ΔΔCt^.

### 4.4 Antibodies

The antibodies and their concentrations used for Western blotting are as follows: anti‐mAcon1 mouse (1 µg/ml; ab83528, Abcam), anti‐ATG8A rabbit (1:1000; ab109364, Abcam), anti‐β‐actin rabbit (1:1000; 4967S, Cell Signaling), anti‐mouse goat IgG‐horseradish peroxidase (HRP) (1:10000; sc2005, Santa Cruz Biotechnology), and anti‐rabbit goat IgG‐HRP (1:10000; sc2004, Santa Cruz Biotechnology) antibodies. The antibodies and their concentrations used for IHC are as follows: anti‐mAcon1 mouse (1:100; ab83528, Abcam), anti‐BRP mouse (1:25; DSHB), anti‐ATG8A rabbit (1:200; ab109364, Abcam), anti‐GFP rabbit (1:1000; Invitrogen), anti‐GFP mouse (1:500; GFP‐G1, DSHB), anti‐mouse goat (1:1000; Alexa Fluor^®^ 488, Invitrogen), anti‐rabbit goat (1:1000; Dylight^®^ 488, Invitrogen), and anti‐rabbit goat (1:1000; Alexa Fluor^®^ 594, Invitrogen) antibodies.

### 4.5 Protein extraction and Western blot analysis

Twenty *Drosophila* heads per sample were homogenized in lysis buffer (with protease inhibitors). The extracted protein from 4–6 samples was used once per experiment. The amount of protein was quantified using a bicinchoninic acid assay kit (Biovison). Protein samples with the same concentration were loaded onto a 15% gel (15 µl each) and electroblotted onto nitrocellulose membranes (IPVH00010; Millipore). The blots were then probed with antibodies against β‐actin (loading control), mAcon1, and ATG8a. Immunoblots were scanned using the ChemiDoc™ Touch Gel Imaging System (#1708370; Bio‐Rad), and bands were analyzed using the Image Lab software (Bio‐Rad). The relative amounts of mAcon1 and ATG8a proteins were normalized to β‐actin in each sample.

### 4.6 Adult *Drosophila* brain dissection and IHC

Adult brains were dissected in phosphate‐buffered saline (PBS) containing ice‐cold 2% paraformaldehyde for 2 h and immediately fixed in PBS containing 4% paraformaldehyde for 1 h at 25°C. After fixation, the brains were kept under vacuum for 1 h in 4% paraformaldehyde in 2% PBT (PBS containing 2% Triton X‐100) and washed 4 times for 10 min each time with 2% PBT. After incubating for 2 h in 2% PBT containing 10% normal goat serum (NGS), the brains were incubated with a primary antibody diluted in 2% PBT solution with 10% NGS and 0.1% sodium azide for 48 h at 4°C. The brains were then washed 4 times with 2% PBT at 25°C and incubated with the secondary antibody for 2 h at 25°C or overnight at 4°C. After washing 4 times for 10 min with 2% PBT, and 3 times for 5 min with PBS, the brains were mounted on a glass slide and coverslipped using Vectashield^®^ (Vector Laboratories).

### 4.7 Image acquisition and analysis

Conventional confocal images were obtained using LSM 700 or LSM 800 confocal microscope (Carl Zeiss). The entire brain image was obtained at a magnification of ×200, and KCs were scanned at a magnification of ×630. All images were analyzed using the ZEN 2 software. The pinhole was set to 1 AU, the laser power was set to the lowest level to visualize the signal clearly, and ×2 line averaging was used. The resolution was scanned at 1024 × 1024 pixels. Z‐stack images were scanned with a side pixel size of 1 AU and approximately 10 sheets.

A previously published method was slightly modified to obtain the ATeam1.03NL signal from KCs (Tsuyama et al., 2013). Briefly, after dissection of the adult brain, the temperature was maintained at 25°C and scanned using a 405‐nm laser in an LSM 800 confocal microscope. Dual emission rate imaging of ATeams was performed using two emission filters (HQ 485/20 for CFP and HQ550/20 for YFP‐FRET). For image analysis, the average intensities of CFP and YFP‐FRET within the region of interest were separately obtained using the ZEN 2 software, and the representative value was obtained by subtracting the background intensity.

Following a previous report (Sun et al., 2015), mt‐Keima signals were obtained to observe mitophagy in the KCs. The fluorescence of mt‐Keima was imaged with two sequential excitations (458 nm, green; 561 nm, red) using a 570–695‐nm emission filter. The mt‐Keima signal obtained using the Zeiss ZEN software was calculated in pixels. Mitophagy levels are defined as the number of pixels with a high red/green ratio divided by the total number of pixels.

### 4.8 Measurement of metabolites by liquid chromatography/mass spectrometry

Forty *Drosophila* heads were collected in liquid nitrogen and homogenized in 300 µL 4:4:2 (v/v) acetonitrile:methanol:water extraction solution at 4°C. After adding 900 μl of the extraction solution, the sample was incubated on ice for 2 h. The homogenates were centrifuged for 10 min at 12,000 rpm to remove debris. The supernatant, dried in a freeze dryer (FDU‐2110, EYELA), was analyzed at the National Instrument Center for Environmental Management in Seoul National University (https://nicem.snu.ac.kr/).

### 4.9 Statistical analyses

All data were tested for normality using SPSS Statistics 25 (IBM) and analyzed using the appropriate statistical method in GraphPad Prism 6 (GraphPad software). Two groups were compared using the two‐tailed unpaired Student's *t* test; multiple groups were compared using one‐ or two‐way analysis of variance (ANOVA). If significance was found in ANOVA, Tukey's or Sidak's multiple comparison test was performed as *post hoc* analysis. Survival curves were analyzed using the Wilcoxon test. Statistical significance was determined when the *p*‐value was <0.05. All data are presented as the mean ±standard error of mean. Asterisks indicate the degree of significance (ns >0.05, *p < 0.05, **p < 0.01, and ***p < 0.001).

## CONFLICT OF INTEREST

The authors declare no conflicts of interest.

## AUTHOR CONTRIBUTIONS

Cho and Park conceived the project. Cho performed the experiments and analyzed the data. Cho and Kim evaluated the data. Park provided the necessary resources. Cho, Kim, and Park wrote the manuscript.

## Supporting information

Fig S1‐S11

Table S1‐S2

## Data Availability

The data that support the findings of this study are available from the corresponding author upon reasonable request.
