# Supplementary material for: Mitochondrial aconitase 1 regulates age‐related memory impairment via autophagy/mitophagy‐mediated neural plasticity in middle‐aged flies
Source: Aging Cell. 2021 Nov 19;20(12):e13520. doi: 10.1111/acel.13520 (PMC8672789; doi:10.1111/acel.13520)

Supplementary Fig. 1

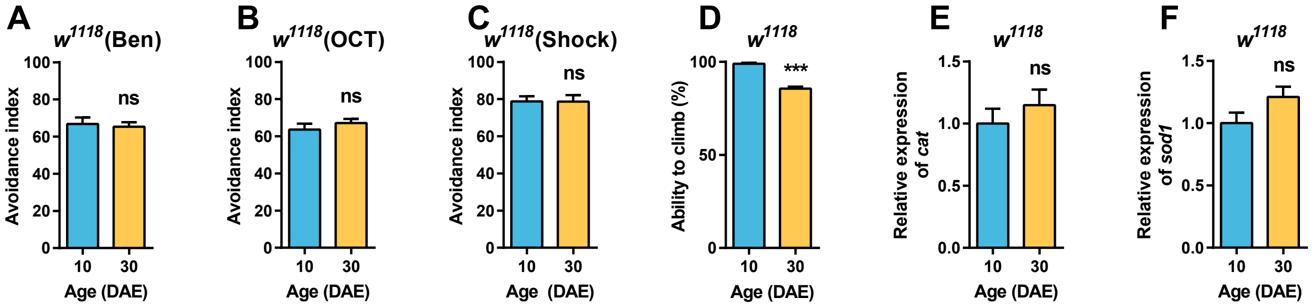

Supplementary Fig. 2

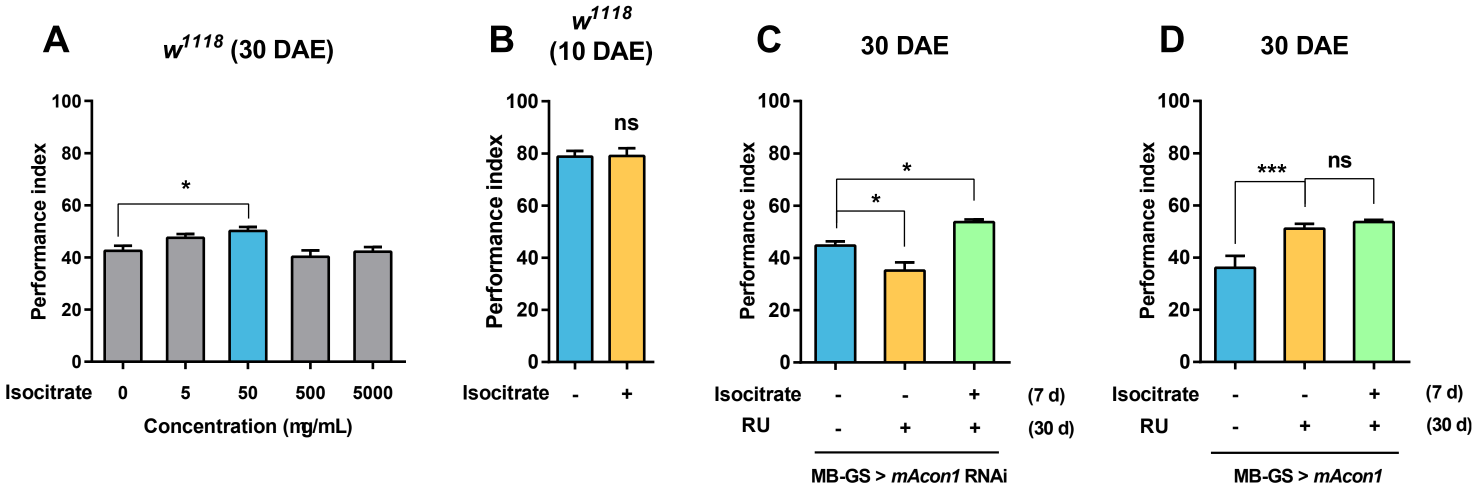

Supplementary Fig. 3

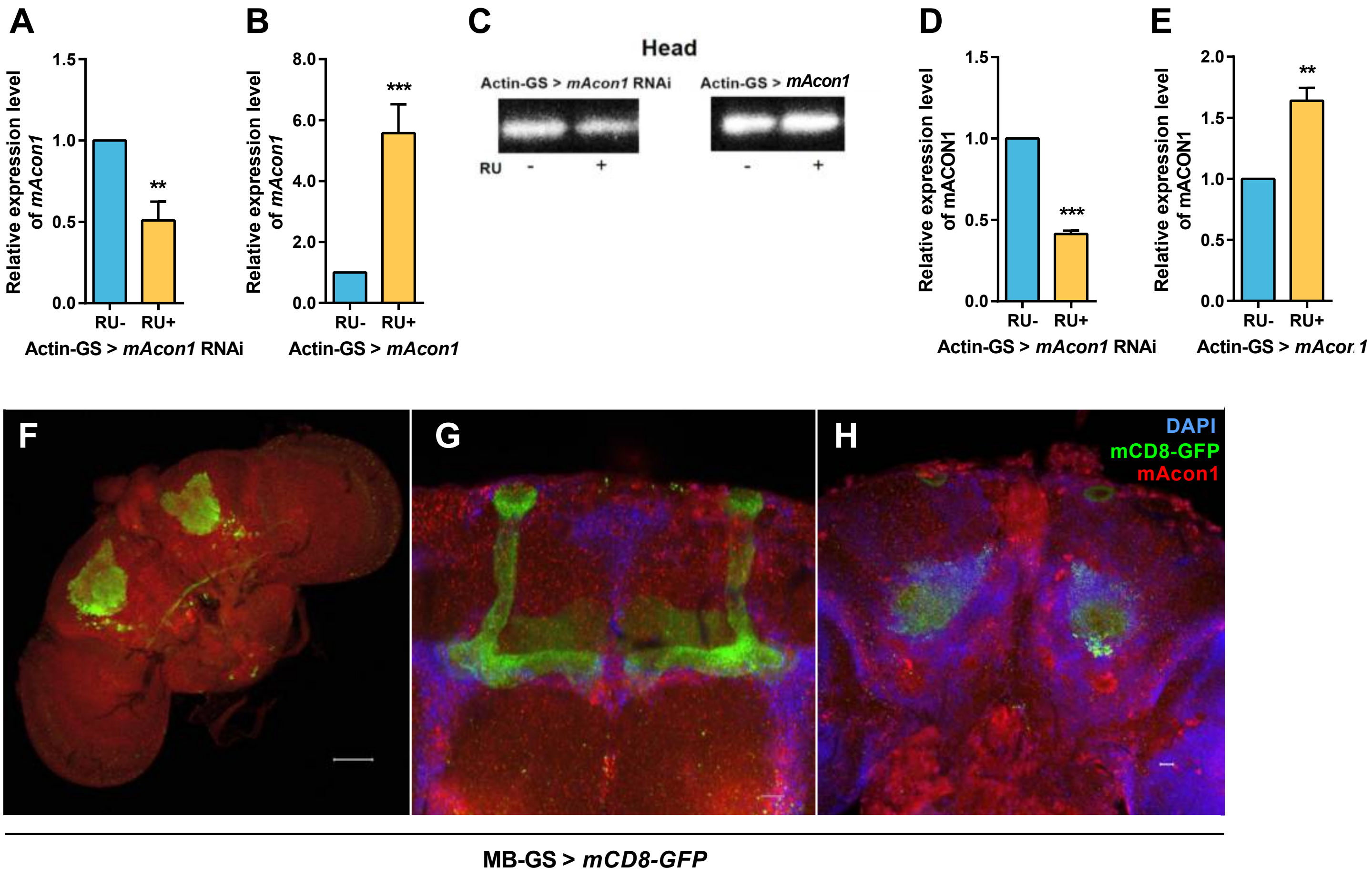

Supplementary Fig. 4

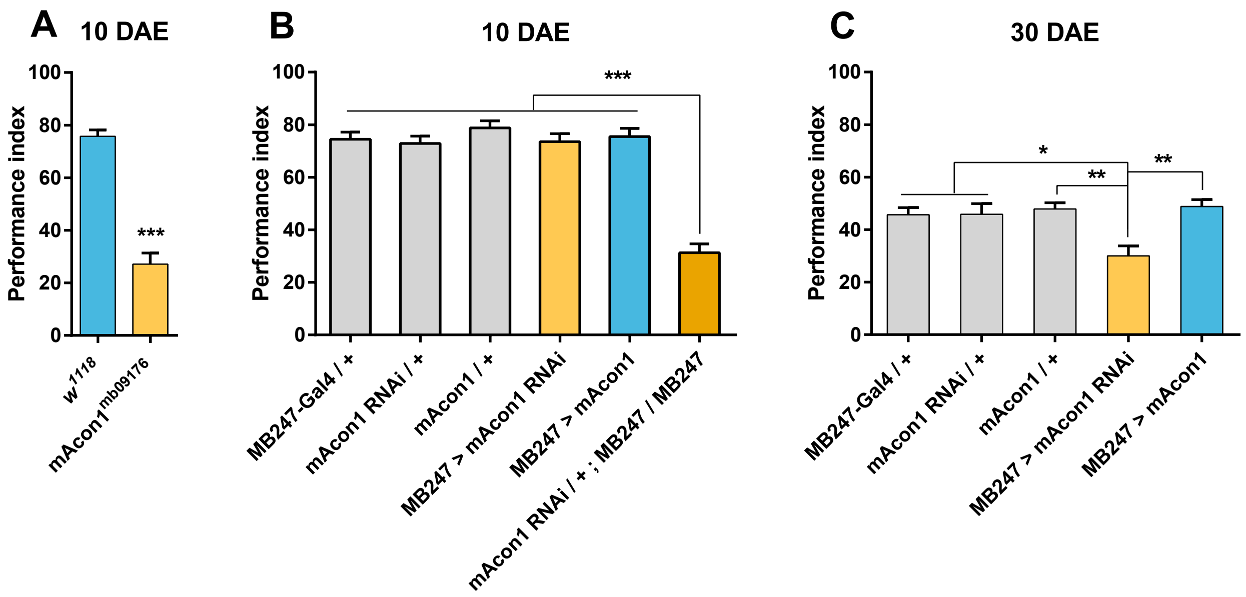

Supplementary Fig. 5

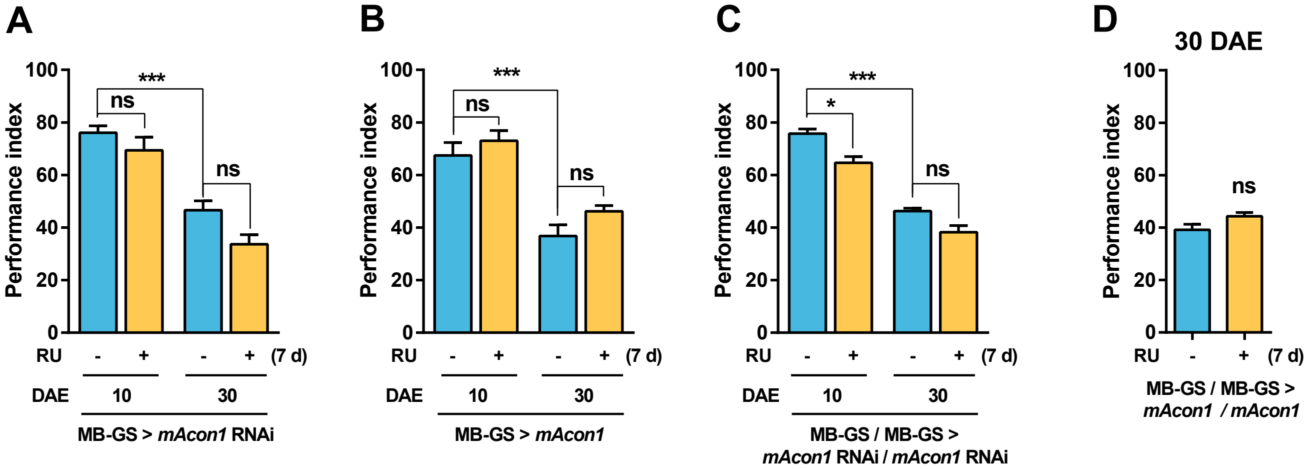

Supplementary Fig. 6

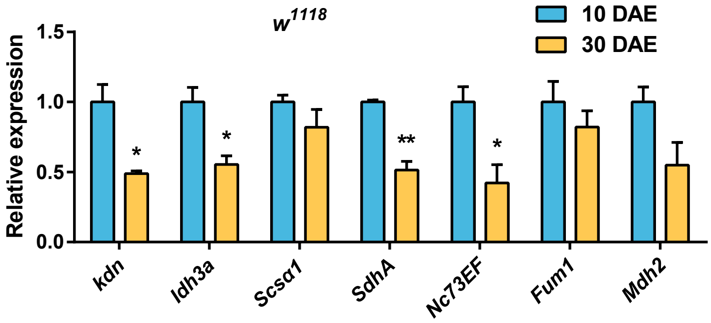

Supplementary Fig. 7

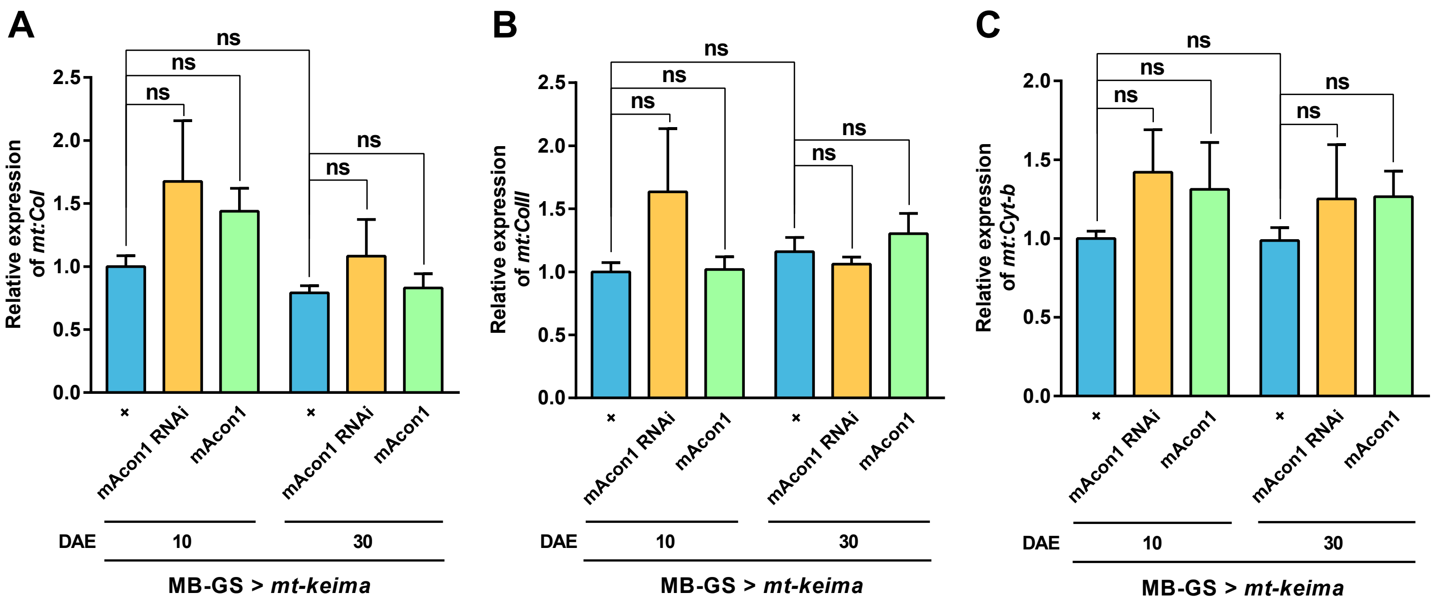

Supplementary Fig. 8

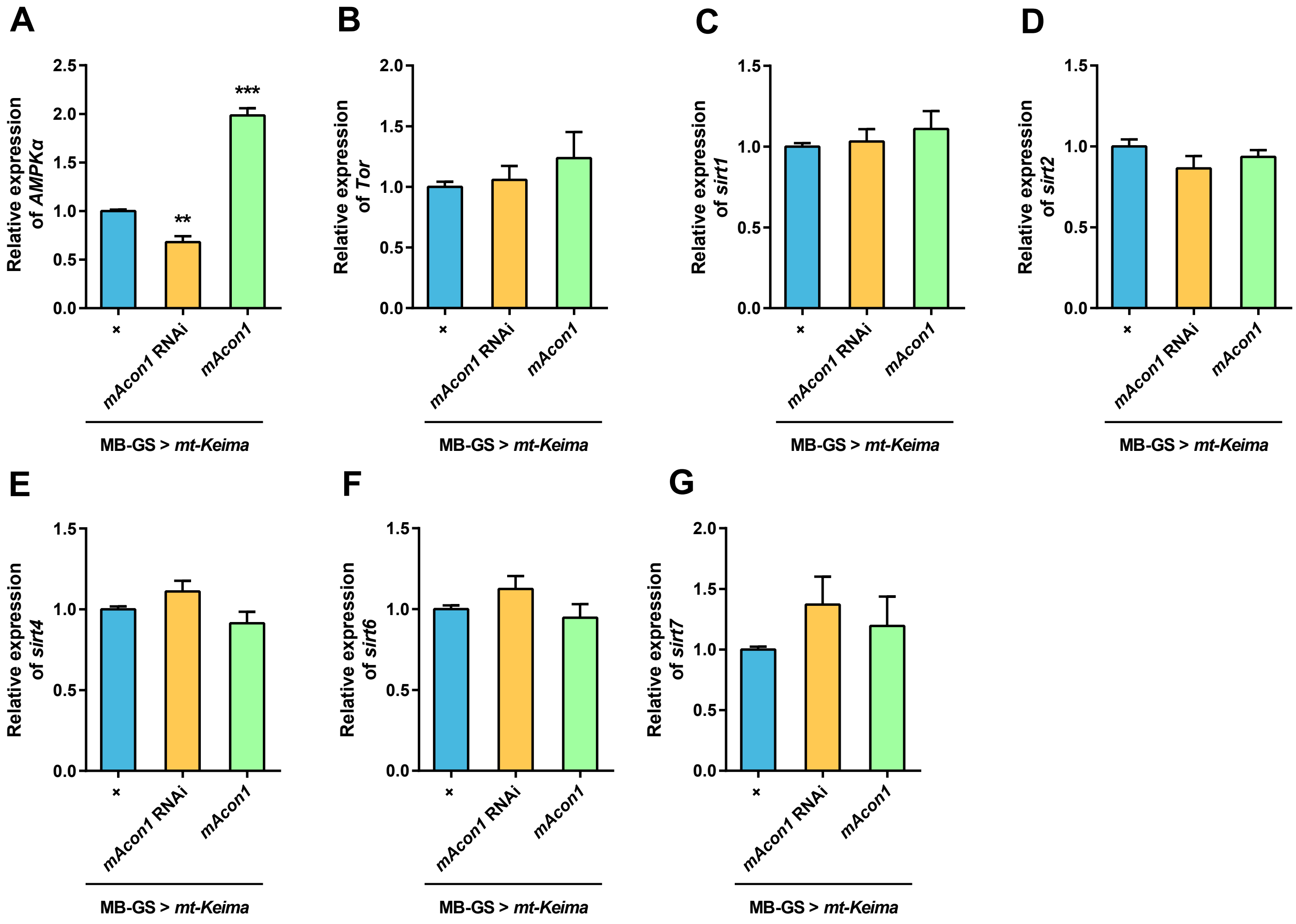

Supplementary Fig. 9

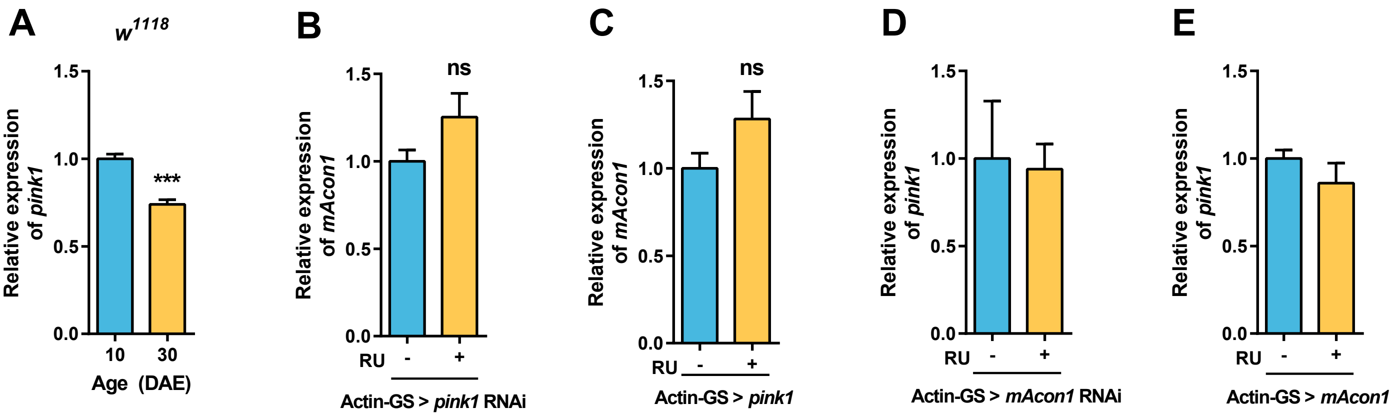

Supplementary Fig. 10

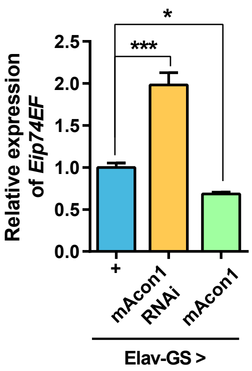

Supplementary Fig. 11

A

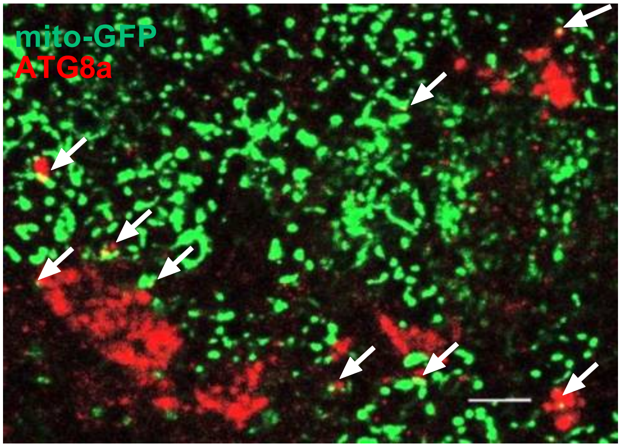

mito-GFP / mAcon1 ; MB-GS / + (10 DAE)

B

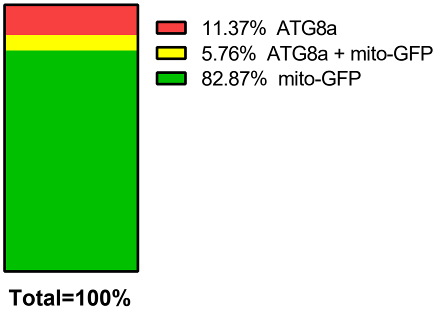

Supplement: Supplementary file 1 — Fig S1‐S11 [file ACEL-20-e13520-s002.pdf]
