# Supplementary material for: Mitochondrial aconitase 1 regulates age‐related memory impairment via autophagy/mitophagy‐mediated neural plasticity in middle‐aged flies
Source: Aging Cell. 2021 Nov 19;20(12):e13520. doi: 10.1111/acel.13520 (PMC8672789; doi:10.1111/acel.13520)
Supplement: Supplementary file 2 — Table S1‐S2 [file ACEL-20-e13520-s001.docx]

**Supporting Information**

**Supplementary Table 1**

| **Genotype (age, treatment)** | **BEN** | **OCT** | **Electric shock** |
| --- | --- | --- | --- |
| *mAcon1* RNAi/+; elav-GS/+ (10 DAE, RU-) | 63.5 ± 2.6 | 63.8 ± 3.5 | 72.0 ± 3.0 |
| *mAcon1* RNAi/+; elav-GS/+ (10 DAE, RU+) | 58.7 ± 3.3 | 60.2 ± 3.4 | 70.4 ± 2.5 |
| *mAcon1* RNAi/+; elav-GS/+ (30 DAE, RU-) | 67.7 ± 2.6 | 60.1 ± 2.7 | 69.3 ± 2.9 |
| *mAcon1* RNAi/+; elav-GS/+ (30 DAE, RU+) | 63.6 ± 2.8 | 55.7 ± 3.8 | 66.0 ± 3.3 |
| *mAcon1*/+; elav-GS/+ (10 DAE, RU-) | 56.4 ± 3.2 | 63.3 ± 4.1 | 79.8 ± 5.2 |
| *mAcon1*/+; elav-GS/+ (10 DAE, RU+) | 55.4 ± 2.9 | 58.2 ± 1.8 | 81.0 ± 2.8 |
| *mAcon1*/+; elav-GS/+ (30 DAE, RU-) | 53.3 ± 4.6 | 51.6 ± 4.7 | 68.0 ± 2.7 |
| *mAcon1*/+; elav-GS/+ (30 DAE, RU+) | 56.4 ± 3.4 | 53.6 ± 3.8 | 70.3 ± 2.7 |
| *mAcon1* RNAi/+; MB-GS/+ (10 DAE, RU-) | 66.9 ± 2.4 | 61.0 ± 5.8 | 65.3 ± 5.5 |
| *mAcon1* RNAi/+; MB-GS/+ (10 DAE, RU+) | 65.4 ± 2.2 | 61.6 ± 2.0 | 67.0 ± 4.0 |
| *mAcon1* RNAi/+; MB-GS/+ (30 DAE, RU-) | 70.9 ± 3.5 | 43.5 ± 4.7 | 59.5 ± 5.0 |
| *mAcon1* RNAi/+; MB-GS/+ (30 DAE, RU+) | 63.8 ± 2.8 | 55.9 ± 3.4 | 58.6 ± 4.1 |
| *mAcon1*/+; MB-GS/+ (10 DAE, RU-) | 64. ± 3.4 | 62.5 ± 2.4 | 64.0 ± 5.0 |
| *mAcon1*/+; MB-GS/+ (10 DAE, RU+) | 55.1 ± 8.9 | 62.6 ± 3.2 | 60.3 ± 4.0 |
| *mAcon1*/+; MB-GS/+ (30 DAE, RU-) | 54.5 ± 4.5 | 55.4 ± 2.9 | 46.9 ± 2.4 |
| *mAcon1*/+; MB-GS/+ (30 DAE, RU+) | 55.2 ± 2.9 | 51.1 ± 2.3 | 44.5 ± 2.5 |
| *mAcon1* RNAi/*mAcon1* RNAi; MB-GS/MB-GS  (10 DAE, RU-) | 51.3 ± 4.0 | 48.5 ± 4.5 | 67. ± 4.5 |
| *mAcon1* RNAi/*mAcon1* RNAi; MB-GS/MB-GS  (10 DAE, RU+) | 54.5 ± 4.0 | 52.0 ± 5.9 | 64.7 ± 4.5 |
| *mAcon1* RNAi/*mAcon1* RNAi; MB-GS/MB-GS  (30 DAE, RU-) | 37.4 ± 2.8 | 38.3 ± 2.5 | 66.5 ± 2.6 |
| *mAcon1* RNAi/*mAcon1* RNAi; MB-GS/MB-GS  (30 DAE, RU+) | 39.5 ± 4.4 | 39.5 ± 3.1 | 68.0 ± 4.1 |
| *mAcon1*/ *mAcon1*; MB-GS/ MB-GS (10 DAE, RU-) | 37.8 ± 3.4 | 51.1 ± 4.8 | 73.5 ± 1.8 |
| *mAcon1*/ *mAcon1*; MB-GS/ MB-GS (10 DAE, RU+) | 36.1 ± 2.9 | 58.4 ± 5.5 | 75.4 ± 2.6 |
| *mAcon1*/ *mAcon1*; MB-GS/ MB-GS (30 DAE, RU-) | 39.1 ± 3.2 | 41.4 ± 5.3 | 72.6 ± 3.0 |
| *mAcon1*/ *mAcon1*; MB-GS/ MB-GS (30 DAE, RU+) | 42.5 ± 3.5 | 42.2 ± 4.7 | 73.0 ± 2.9 |
| *mAcon1* RNAi/+; MB-GS/*pink1* RNAi (30 DAE, RU-) | 54.7 ± 5.3 | 41.5 ± 4.8 | 82.1 ± 1.7 |
| *mAcon1* RNAi/+; MB-GS/*pink1* RNAi (30 DAE, RU+) | 51.6 ± 6.5 | 39.4 ± 5.8 | 81.1 ± 1.9 |
| *mAcon1*/+; MB-GS/*pink1* RNAi (30 DAE, RU-) | 65.5 ± 4.3 | 59.6 ± 5.3 | 72.2 ± 2.6 |
| *mAcon1*/+; MB-GS/*pink1* RNAi (30 DAE, RU+) | 66.4 ± 5.0 | 51.0 ± 5.4 | 73.3 ± 2.1 |
| +/MB247 (10 DAE) | 44.2 ± 5.2 | 48.2 ± 5.8 | 76.9 ± 2.6 |
| *mAcon1* RNAi/+ (10 DAE) | 46.3 ± 6.9 | 52.8 ± 6.1 | 80.2 ± 2.1 |
| *mAcon1*/+ (10 DAE) | 43.6 ± 4.6 | 49.5 ± 5.3 | 79.6 ± 1.9 |
| *mAcon1* RNAi/+; MB247/+ (10 DAE) | 30.8 ± 5.8 | 57.1 ± 7.0 | 79.8 ± 1.5 |
| *mAcon1*/+; MB247/+ (10 DAE) | 42.6 ± 7.8 | 51.8 ± 5.8 | 79.6 ± 2.6 |
| *mAcon1*^mb09176^ / + (10 DAE) | 55.3 ± 4.4 | 53.4 ± 3.8 | 73.9 ± 2.1 |
| *mAcon1* RNAi/+; MB247/MB247 (10 DAE) | 58.0 ± 2.9 | 53.8 ± 5.8 | 79.3 ± 2.1 |

**Suppl. Table 1:** Aversive olfactory and electric shock avoidance scores for different genotypes and ages used in this study. None of the experimental groups showed significantly impaired aversion to the odors compared to their corresponding controls.

**Supplementary Table 2**

| Gene symbol | Sequence | |
| --- | --- | --- |
|  | 5’ | 3’ |
| *sod1* | GGACCGCACTTCAATCCGTA | TTGACTTGCTCAGCTCGTGT |
| *cat* | GATGCGGCTTCCAATCAGTTG | GCAGCAGGATAGGTCCTCG |
| *mAcon1* | GCTGCGAGATTGATGAACGC | AACTTGGACAGAGCCACTTTG |
| *kdn* | TGCCAAATGTGGGAGCCTATG | ATGCTGCTTGCGGAAGTTCTT |
| *idh3a* | AAGGGTCCTCTGATGACGC | CCTCGGTGTTCTCACGGAT |
| *nc73ef* | TCCGCAAGCGTTTTGAGAC | TCCGTTGATACGTCGATGATCT |
| *scsα1* | TCATGCCGGGTCACATTCAC | TCGATGAAGTCGGTTCCGTTG |
| *sdha* 5 | ATGTCCCTCTCGTTGCTTCTG | GAGTGATGCGAGCCGACTTA |
| *fum1* | TGAACAAGGAGTTCGGACTGG | AGTGGTCGTCGTATAGCTTGC |
| *mdh2* | CGCCGATCTGTCGCATATC | CACCGGGTTGGTGATGATGG |
| *mt:CoI* | ATTGGCTGGAATACCTCGACG | ATGTTCAGCTGGCGGAGTAT |
| *mt:Cyt-b* | ACTCCTTTAGTAACACCTGCCC | TGGTCGAGCTCCAATTCAAGT |
| *eip74EF* | CATACTAGTTGCCGGCGTAT | TTTAGCACTTCCCACTCCTG |
| *ampka* | TGGGCACTACCTACTGGG | ATCTGGTGCTCGCCGATCTT |
| *tor* | TTACTGCCAGGAAGGGCATTT | TGACGGACACATCGTTGATTAG |
| *sirt1* | CATTATGCCGCATTTCGCCA | GAAGGTGTTCACTGAGGCCA |
| *sirt2* | TCCCCAGGTTCCGGGTTATAC | AAACGGCGCTGGATTCTTTTC |
| *sirt4* | CGGATCGATCGTCACGAGTT | CTGCGTACACTCGGGTATCC |
| *sirt6* | TCTTCCCGAAAACGACCTCG | CAAAGTGGTTCCAAGCGCAA |
| *sirt7* | AACGCGGGCCAAGATGAAT | GCGCATGGAGTCGCATTTTC |
| *pink1* | CATAGCCAAAGGTTGTGC | ATCCGAGGCAACATCTTTCTTGA |
| *rp49* | TCCTACCAGCTTCAAGATGAC | CACGTTGTGCACCAGGAACT |

**Suppl. Table 2:** Sequences of primers used for RT-PCR. The mRNA levels of each sample were normalized to that of *rp49*, which did not change with age.

**Supplementary Figure Legends**

**Suppl. Fig. 1.** Comparison of the sensory capacity of the *w^1118^* young and middle-aged flies

No significant differences are observed in the odor avoidance index (**A**, benzaldehyde, BEN; **B,** octanol, OCT) and electric shock avoidance index (**C**, 60 V) between the young and middle-aged flies (n = 8; using *t*-test). (**D**) Anti-geotactic locomotor activity of the *w^1118^* flies decreases slightly but significantly in middle-aged flies (n = 8; using *t-*test). (**E and F**) In young and middle-aged flies, no significant differences are observed between the mRNA levels of catalase (*cat*) and superoxide dismutase 1 (*sod1*), levels of which are known to decrease as aging progresses (n = 9–12; using *t*-test).

**Suppl. Fig. 2.** Effects of short-term isocitrate feeding on AMI

(**A**) In *w^1118^* middle-aged flies, AMI significantly alleviates when the flies are fed 50 µg/mL isocitrate for 30 d (F_(4, 35)_ = 4.986, p < 0.01, using one-way ANOVA; *post-hoc* analysis using Sidak’s test). (**B**) Learning measured after feeding the *w^1118^* young flies with 50 µg/mL isocitrate for 7 d (n = 8; using *t*-test). (**C–D**) AMI measured after feeding 50 µg/mL isocitrate for 7 d to middle-aged flies that regulated *mAcon1* expression in KCs in adulthood. AMI alleviates when the expression of *mAcon1* either decreases (**C**, F_(2, 29)_ = 19.46) or increases (**D**, F_(2, 29)_ = 13.65) (p < 0.001 using one-way ANOVA; *post-hoc* analysis using Tukey’s test for both).

**Suppl. Fig. 3.** Expression patterns of *mAcon1* RNAi and UAS-*mAcon1* lines

Expression of *mAcon1* is regulated in all cells under the control of *actin*-GS Gal4 driver (feeding 150 µM RU for 10 d). *mAcon1* mRNA, and protein levels measured in the head of flies (**A**, *actin*-GS > *mAcon1* RNAi; **B**, *actin*-GS > *mAcon1*; n = 6; using *t-*test). (**D–E**) IHC was performed using 4′,6-diamidino-2-phenylindol (blue), GFP-specific antibody (green), and *mAcon1*-specific antibody (red) in the adult brain expressing GFP (MB-GS > mCD8-GFP), which was used to label cell membranes of the KCs (scale bar, 10 µm).

**Suppl. Fig. 4.** Learning of *mAcon1* mutant flies and flies where *mAcon1* expression is regulated in KCs

When *mAcon1* expression is controlled in KCs using MB247-Gal4, learning in young flies is not affected. However, the learning of young *mAcon1-*mutant flies (*mAcon1^mb09176^*) decreases significantly, compared to that in the young control flies. Increasing the RNAi intensity by doubling the MB247-Gal4 driver results in a significant reduction in learning in young flies, compared to the control groups (F_(7, 56)_ = 47.84, p < 0.001 using one-way ANOVA; *post-hoc* analysis using Tukey’s test).

**Suppl. Fig. 5.** Short-term effects of *mAcon1* expression in KCs on learning

(**A and B**) When *mAcon1* expression is controlled in KCs by RU feeding for 7 d, learning or AMI does not differ significantly irrespective of increased or decreased *mAcon1* expression (**A**, age [F_(1, 28)_ = 72.93, p < 0.001], and RU effects [F_(1, 28)_ = 6.649, p < 0.05] using two-way ANOVA; *post-hoc* analysis using Tukey’s test; **B**, age effect (F_(1, 28)_ = 53.61, p < 0.001) using two-way ANOVA; *post-hoc* analysis using Tukey’s test). (**C and D**) Under the same activation conditions, increasing the intensity of *mAcon1*-knockdown or overexpression by making homozygous transgenic flies does not change the learning of young flies or AMI in middle-aged flies (**C**, age [F_(1, 28)_ = 186.3, p < 0.001], and RU effects [F_(1, 28)_ = 22.06, p < 0.001] using 2-way ANOVA; *post-hoc* analysis using Tukey’s test; **D**, n = 8; using *t*-test).

**Suppl. Fig. 6.** Enzyme-coding genes with significantly reduced expression in the middle-aged flies compared to the young *w^1118^* are *kdn* (citrate-synthase), *Idh3a* (isocitrate dehydrogenase), and *SdhA* (succinate dehydrogenase). The expression levels of *Scsα1* (succinyl-CoA hydrolase), *Fum1* (fumarate hydratase), and *Mdh2* (malate dehydrogenase) are not different.

**Suppl. Fig. 7.** *mAcon1* expression levels do not alter the content of mitochondrial DNA

(**A–C**) Mitochondrial DNA (*mt:CoI*, *mt:CoIII*, and *mt:cyt-b*), isolated using FACS, was measured in KCs expressing mt-Keima. Although the statistical analysis of *mt:CoI* data indicates an age effect (**A**, age effect [F_(1, 27)_ = 5.741, p < 0.05] two-way ANOVA), no significant differences are found among the treatment groups of *mAcon1* expression levels or between the age groups of *mt:CoI*, *mt:ColII*, and *mt:cyt-b*.

**Suppl. Fig. 8.** mRNA expression levels of mitophagy-related genes in KCs after *mAcon1* expression is regulated

Although *mAcon1* expression is regulated in the KCs of young flies, mt-Keima protein is also expressed in KCs. RT-PCR analysis of mitophagy-related genes (*AMPKα*, *Tor*, and *sirt1-7*) was performed with KCs collected using FACS. Only *AMPKα* expression is significantly reduced by *mAcon1*-knockdown or increased by *mAcon1*-overexpression (**A**, F_(2, 12)_ = 142.2, p < 0.001 using one-way ANOVA; *post-hoc* analysis using Tukey’s test; **C–G**). Abbreviation: Sirt, Sirtuins.

**Suppl. Fig. 9.** Aging results in significant reduction of *pink1* mRNA expression

(**A**) *pink1* mRNA expression in the heads of the *w^1118^* middle-aged flies is significantly lower than that in young flies (n = 6; using *t*-test). (**B–E**) *pink1* expression levels do not affect *mAcon1* expression levels in fly heads, and *vice versa* (n = 6–10; using *t*-test).

**Suppl. Fig. 10.** Expression of *Eip74EF*, the target gene of nejire, is regulated according to the expression of *mAcon1*.

Neuronal overexpression of *mAcon1* increases the expression level of *Eip74EF*. Knockdown of *mAcon1* decreases *Eip74EF* expression (F_(2, 15)_ = 54.65, p < 0.001).

**Suppl. Fig. 11.** Immunohistochemical visualization of mitochondria and autophagosomes in KCs

Mitochondria are visualized using mito-GFP. Autophagosomes are visualized immunohistochemically using specific antibodies to ATG8α protein, which comprises a part of autophagosomes in KCs. (**A**) Mito-GFP (green) and ATG8α (red) are visualized. Some colocalized signals are indicated with arrows (yellow). Scale bar: 10 µm. (**B**) The intensity of each color channel is converted and quantified based on the number of pixels.
